# Sociodemographic and work-related factors associated with psychological resilience in South African healthcare workers: a cross-sectional study

**DOI:** 10.1186/s12913-024-11430-0

**Published:** 2024-08-24

**Authors:** Thandokazi Mcizana, Shahieda Adams, Saajida Khan, Itumeleng Ntatamala

**Affiliations:** 1 Division of Actuarial Science, School of Management Studies, University of Cape Town, Cape Town, South Africa; 2 Division of Occupational Medicine and Centre for Environmental and Occupational Health Research, School of Public Health, University of Cape Town, Cape Town, South Africa; 3 Livingstone Tertiary Hospital, Department of Health, Gqeberha, South Africa; 4 Faculty of Health Science, Nelson Mandela University, Gqeberha, South Africa

**Keywords:** Resilience, Healthcare workers, Ambulance personnel, Occupational, Doctors

## Abstract

**Background:**

Psychological resilience facilitates adaptation in stressful environments and is an important personal characteristic that enables workers to navigate occupational challenges. Few studies have evaluated the factors associated with psychological resilience in healthcare workers.

**Objectives:**

To determine the prevalence and factors associated with psychological resilience in a group of South African medical doctors and ambulance personnel.

**Materials and methods:**

This analytical cross-sectional study used secondary data obtained from two studies conducted among healthcare workers in 2019 and 2022. Self-reported factors associated with resilience, as measured by the Connor-Davidson Resilience Scale-10 (CD-RISC-10), were evaluated. R statistical software was used for analysing the data and performing statistical tests.

**Results:**

A total of 647 healthcare workers were included in the study, of which 259 were doctors and 388 were ambulance personnel. Resilience scores were low overall (27.6 ± 6.6) but higher for ambulance personnel (28.0 ± 6.9) than for doctors (27.1 ± 6.0) (*p* = 0.006). Female gender (OR 1.94, 95%CI 1.03–3.72, *p* = 0.043), job category (OR 6.94 95%CI 1.22–60.50, *p* = 0.044) and overtime work (OR 13.88, 95%CI 1.61–368.00, *p* = 0.044) significantly increased the odds of low resilience for doctors. Conversely, salary (OR 0.13, 95%CI 0.02–0.64, *p* = 0.024) and current smoking status (OR 0.16, 95%CI 0.02–0.66, *p* = 0.027) significantly reduced the odds of low resilience amongst doctors. In addition, only previous alcohol use significantly reduced the odds of low resilience for ambulance personnel (OR 0.44, 95%CI 0.20–0.94, *p* = 0.038) and overall sample (OR 0.52, 95%CI 0.29–0.91, *p* = 0.024).

**Conclusions:**

Resilience was relatively low in this group of South African healthcare workers. The strong association between low resilience and individual and workplace factors provides avenues for early intervention and building resilience among healthcare workers.

**Supplementary Information:**

The online version contains supplementary material available at 10.1186/s12913-024-11430-0.

## Introduction

The healthcare systems of most low- and middle-income countries (LMICs) are under severe strain due to high patient load, significant burden of communicable and noncommunicable diseases, lack of human and financial resources, the brain drain phenomenon, corruption and poor administration [1–4]. South Africa, an upper middle-income country, faces similar challenges, with a quadruple burden of disease including HIV/AIDS and tuberculosis, high maternal and child mortality, high levels of violence and injuries and noncommunicable diseases [5]. Poor health outcomes and a disproportionate distribution of healthcare resources in the country may be ascribed to the legacy of an undemocratic political apartheid regime (1948–1993) compounded by ongoing challenges in managing the health system in a post-apartheid South Africa [4, 5]. In 2021, for example, South Africa had a doctor-patient ratio of 80 physician per 100,000 people in South Africa, which is lower than the average in upper middle-income countries of 210 physicians per 100,000 people [6]. South Africa’s government is currently in the process of implementing a National Health Insurance (NHI) scheme to address the tremendous challenges that plague the health system [2]. However, the country’s preparedness remains uncertain, especially given the ongoing shortage of healthcare worker posts and rising unemployment in the health sector [5, 7]. These challenges place immense pressure on employed healthcare workers, making psychological resilience an important inherent ability that can aid in supporting and protecting healthcare workers against adverse mental health outcomes and contributing to improved service delivery.

Psychological resilience is an important personal characteristic that enables healthcare workers to navigate the challenges encountered in their occupation [8]. Herrman and colleagues explored the evolution of the term in their narrative review and concluded that fundamentally, resilience is the ‘inherent ability’ for one to adapt positively following adversity or stressful events [9]. As such, psychological resilience describes an individual’s coping mechanism, optimism, self-efficacy, high levels of hope and thriving mental health amid adversity and challenging circumstances [10]. Research on the role of psychological resilience as a protective factor in frontline healthcare workers has increased recently during the coronavirus disease (COVID-19) pandemic [11]. Much of the research in this area has been conducted in high-income countries (HICs) and China, and little is known about the factors that predict psychological resilience in workers in LMICs, including South Africa [11]. A systematic review on resilience among primary healthcare workers, found that most research on the topic primarily frames resilience as an explanatory variable in relation to burnout [12]. This study therefore aimed to determine the prevalence, and factors associated with psychological resilience of healthcare workers practising in the South African healthcare system.

## Methods

### Study design and setting

This is an analytical cross-sectional study using secondary data obtained from two cross-sectional studies of healthcare workers in South Africa. The first study on post-traumatic stress disorder (PTSD) included ambulance personnel employed by the Western Cape Department of Health, and data was collected between 15 November 2019 and 17 January 2020 [13]. This study included 388 responses out of approximately 2000 ambulance personnel. The second study on burnout included medical doctors employed in three public sector hospitals in the Eastern Cape province, and data was collected between 1 April and 31 May 2022 [14]. This study included 260 responses out of 430 doctors. The present study included data of all healthcare workers who had completed the Connor-Davidson Resilience Scale-10 (CD-RISC-10) questionnaire and relevant sociodemographic and occupational questions.

### Measurements

This study used secondary data generated from self-administered questionnaires that consisted of sociodemographic factors, work-related factors, and the CD-RISC-10 questionnaire.

#### Sociodemographic and work-related factors

The data obtained from the questionnaires included self-reported information on age, gender, language, marital status, job category, professional qualifications, overtime work, salary, and length of service. In addition, data on mental health and medical history, including self-reported mental health conditions and substance use (smoking, alcohol use, illicit and prescription drugs), year of debut, and the use of substances to manage work-related stress, were obtained.

#### Outcome

Psychological resilience (outcome variable) was measured using the 10-item CD-RISC questionnaire. The CD-RISC-10 is a self-administered 10-item questionnaire, which is a shorter version of the CD-RISC-25. Participants identified their adaptive behaviours in stressful situations and scored them on a 5-point Likert scale (0 = not at all true, 4 = true nearly all the time) [15]. The resulting scores ranged between 0 and 40. This scale has previously been reported to be a reliable and efficient measure of psychological resilience for adults [16]. In addition, it has previously been validated for use in South Africa by Pretorius and Padmanabhanunni as a measure of psychological resilience and has been used in several studies of South African healthcare workers [3, 13, 14, 17–19]. Written permission to use the scale was previously obtained [13, 14].

### Data analysis

After ethical approval, the secondary data were received and cleaned in password-protected Microsoft Excel. R statistical software (version 4.3.1) was used for analysing the data and performing the statistical tests. Descriptive statistics for continuous variables in this study are presented as the means (standard deviations) and medians (interquartile ranges) where appropriate. In addition, descriptive statistics for categorical variables are presented as proportions.

Mann‒Whitney and Kruskal‒Wallis tests were used to determine significant differences in CD-RISC-10 scores. In addition, unadjusted logistic regression and adjusted logistic regression (adjusted for age and gender) were performed. Low resilience, as an outcome measure, was defined as a CD-RISC-10 score less than 25.5 [20]. Variables from the adjusted logistic regression analysis with a p value less than 0.250 were selected for the multivariable logistic regression model to investigate factors associated with increased resilience score. The odds ratios (OR), 95% confidence intervals (95%CI) and p values (p) were calculated for both the univariable and multivariable analyses. A p value of less than 0.050 was considered the cut-off point for statistical significance.

#### Missing data

Only the age factor had missing data of more than 1% of the total recorded values and thus necessitated imputation (see Supplementary Table S1 and Supplementary Fig. S1 online). Age is also important when performing this regression analysis, as age has previously been reported to be an important confounder of psychological resilience and needs to be adjusted for when performing regression analysis [11, 21–23]. Multiple imputation was chosen because it results in valid statistical inferences [24]. To assess the sensitivity of the results with respect to the multiple imputation method chosen, multiple imputation using the three methods available in the Multivariate Imputation by Chained Equation (MICE) package in R were performed (see Supplementary Table S2 online). The imputed data from the Classification and regression tree (CART) method was chosen for use in the following regression analysis, given its minimal impact on the distribution of the age factor. Supplementary Fig. S2 shows the distribution of the age factor before and after CART imputation.

## Results

From the original datasets received (648 records), only one record was removed because the participant indicated that they were gender nonconforming, resulting in several skewed results. In total therefore, 647 observations were included in the present analysis, of which 259 were from doctors and 388 were from ambulance personnel.

### Sociodemographic and work-related characteristics

Among the 259 doctors, the majority, 150 (57.9%) were female, while most ambulance personnel, 213 (54.9%) were male (Table 1). Most of the doctors, 171 (66.0%) were English speaking and 110 (42.5%) were in the 20–29 years age group, while most of the ambulance personnel, 178 (45.9%) were Afrikaans speaking and, 144 (37.1%) were in the 30–39 years age group. Doctors’ years of service in the current role were lower, with a median of 2 (IQR: 4), while ambulance personnel had a median of 7 (IQR: 9). A greater percentage of doctors, 251 (96.9%) reported working overtime than, 266 (68.6%) ambulance personnel.

### Substance use, mental health, and work-related stress management

The prevalence of smoking was greater among ambulance personnel, 118 (30.4%) than among, 23 (8.9%) of doctors, while current alcohol usage was 166 (64.1%) for doctors, greater than 200 (51.5%) for ambulance personnel (Table 2). Only 18 (2.8%) of the overall sample reported current use of illicit substances or drugs. A quarter of the doctors, 65 (25.1%), reported having been diagnosed with a mental health condition compared to 43 (11.1%) of the ambulance personnel. In addition, 45 (17.4%) of doctors reported being on treatment for a mental health condition, compared to, 28 (7.2%) of ambulance personnel.

Regarding managing work-related stress (WRS), more than a quarter, 103 (26.5%) of the ambulance personnel self-reported the need to smoke to manage WRS, while 53 (20.5%) of the doctors reported the need to use alcohol to manage WRS. Interestingly, 29 (4.5%) of the overall sample felt the need to use illicit drugs to manage WRS, which is higher than the current prevalence of illicit drug use. Most participants supported the provision of psychological counselling, 492 (76.0%) and addressing staff shortages, 483 (74.7%) to assist with reducing WRS.

### Prevalence of resilience

The overall average CD-RISC-10 score was 27.6 (± 6.6) among the 647 healthcare workers in this study (Table 2). The average CD-RISC-10 score for the ambulance personnel was 28.0 (± 6.9), which was significantly higher than the average score of 27.1 (± 6.0) for the doctors (*p* = 0.006). The total score for the CD-RISC-10 can be classified into a 4-level variable using quantiles: lowest (0–24), low (25–28), moderate (29–32), and highest (33–40) [15]. More than half of the doctors (58.7%) were classified as having the lowest or low resilience. However, for ambulance personnel, the majority (54.2%) were classified as having moderate or high resilience.

**Table 1:** Sociodemographic and work-related characteristics

Participant characteristics	Doctors		Ambulance personnel		Overall	
	*N*	%	*N*	%	*N*	%
**Gender**						
Male	109	42.1	213	54.9	322	49.8
Female	150	57.9	175	45.1	325	50.2
**Age**						
20–29	110	42.5	52	13.4	162	25.0
30–39	73	28.2	144	37.1	217	33.5
40–49	50	19.3	106	27.3	156	24.1
> 50	26	10.0	37	9.5	63	9.7
Missing	0	0.0	49	12.6	49	7.6
**Home language**						
English	171	66.0	122	31.4	293	45.3
Afrikaans	54	20.8	178	45.9	232	35.9
IsiXhosa	31	12.0	84	21.6	115	17.8
Other	3	1.2	4	1.0	7	1.1
**Relationship Status**						
Married	117	45.2	174	44.8	291	45.0
Never married	127	49.0	172	44.3	299	46.2
Divorced/Separated/Widowed	15	5.8	42	10.8	57	8.8
**Professional health qualification**						
Yes	259	100.0	322	83.0	581	89.8
No	0	0.0	66	17.0	66	10.2
**Job category**						
Operational services/EMS	0	0.0	277	71.4	277	42.8
Support staff/EMS	0	0.0	111	28.6	111	17.2
Junior doctors	85	32.8	0	0.0	85	13.1
Senior doctors	174	67.2	0	0.0	174	26.9
**Years employed in current role** ^†^	2 (4)		7 (9)		5 (8)	
Missing (%)	0	0.0	5	1.3	5	0.8
**Over-time work**						
Yes	251	96.9	266	68.6	517	79.9
No	8	3.1	122	31.4	130	20.1
**Monthly Salary (ZAR)**						
R0 - R15 000	0	0.0	165	42.5	165	25.5
R15 001 - R30 000	0	0.0	193	49.7	193	29.8
R30 001 - R50 000	88	34.0	30	7.7	118	18.2
> R50 001	171	66.0	0	0.0	171	26.4

^†^ Data are presented as the median (interquartile range)

EMS: Emergency medical services; ZAR/R: South African Rand

**Table 2:** Frequency and distribution of general and mental health-specific variables

Participant characteristics	Doctors		Ambulance personnel		Overall	
	*N*	%	*N*	%	*N*	%
**Age started smoking (m**,** SD)**^†^	20.1	3.7	18.6	4.6	18.9	4.4
**Age started illicit drugs (m**,** SD)**^†^	20.1	3.8	21.4	6.6	21.0	6.0
**Smoking history**						
Never used	213	82.2	235	60.6	448	69.2
Previous smoker	23	8.9	35	9.0	58	9.0
Current smoker^‡^	23	8.9	118	30.4	141	21.8
**Alcohol history**						
Never used	54	20.8	110	28.4	164	25.3
Previous alcohol user	39	15.1	78	20.1	117	18.1
Current drinker ^‡^	166	64.1	200	51.5	366	56.6
**Illicit drug use**						
Never used	239	92.3	342	88.1	581	89.8
Previous illicit drug user	13	5.0	35	9.0	48	7.4
Current illicit drug user ^‡^	7	2.7	11	2.8	18	2.8
**Substance use to manage WRS**						
Feel need to smoke to manage WRS	45	17.4	103	26.5	148	22.9
Feel need to drink alcohol to manage WRS ‡	53	20.5	44	11.3	97	15.0
Feel need to use illicit drugs to manage WRS ‡	13	5.0	16	4.1	29	4.5
**Mental health**						
Ever diagnosed with a mental health condition ‡	65	25.1	43	11.1	108	16.7
Currently on treatment for mental health condition	45	17.4	28	7.2	73	11.3
**Resilience**,** CD-RISC-10 score (m**,** SD)**^†^	27.1	6.0	28.0	6.9	27.6	6.6
Lowest (0–24)	75	29.0	101	26.0	176	27.2
Low (25–28)	77	29.7	77	19.8	154	23.8
Moderate (29–32)	63	24.3	105	27.1	168	26.0
Highest (33–40)	44	17.0	105	27.1	149	23.0
**Which intervention would assist most with reducing WRS?**						
Address staff shortages	240	92.7	243	62.6	483	74.7
Lessen workload	102	39.4	119	30.7	221	34.2
Have more supportive management	171	66.0	242	62.4	413	63.8
Rotate shifts to allow enough rest	115	44.4	82	21.1	197	30.4
Provide psychological counselling	104	40.2	388	100.0	492	76.0

^†^ Data are presented as the mean and standard deviation

^‡^ Missing data (see Supplementary Table S1 online for details)

CD-RISC-10: Connor-Davidson Resilience Scale-10; WRS: work-related stress

### Factors associated with resilience

Bivariable analysis was performed to examine differences in CD-RISC-10 scores across several sociodemographic and work-related variables (Table 3). Compared with female doctors, male doctors had significantly greater resilience scores (*p* < 0.001). Those in certain job categories, such as senior doctors and ambulance personnel, had significantly greater resilience than did junior doctors (*p* = 0.019). In addition, doctors who earned in the highest salary bracket demonstrated greater resilience than did those who earned less (*p* = 0.020). Doctors who were current smokers had greater resilience (30.7) than those who had never smoked (27.2) or were previous smokers (26.7) (*p* = 0.012). In addition, a history of alcohol use significantly increased resilience for ambulance personnel (30.5) compared to current users (27.6) and never users (27.1) (*p* = 0.002). Participants who self-reported as having been diagnosed with a mental health condition had significantly lower resilience scores compared to those who have not, for doctors (*p* = 0.037), ambulance personnel (*p* = 0.010) and overall sample (*p* < 0.001). In addition, ambulance personnel and the overall sample currently on treatment for a mental health condition had significantly lower resilience scores (*p* = 0.029 and *p* = 0.002 respectively). Lastly, participants who felt the need to drink alcohol to manage WRS had significantly lower resilience scores amongst doctors (*p* = 0.034), ambulance personnel (*p* = 0.048) and overall sample (*p* = 0.002).

Unadjusted (see Supplementary Table S3 online) and adjusted (Supplementary Table S4 online) logistic regression analyses were also performed. Table 4 below provides the results from the multivariable logistic regression analysis performed with selected variables with p value less than 0.25 from Supplementary Table S4 online. For doctors, female gender, job category and overtime work significantly increased the odds of low resilience (OR 1.94, 95%CI 1.03–3.72, *p* = 0.043; OR 6.94, 95%CI 1.22–60.50, *p* = 0.044 and OR 13.88, 95%CI 1.61–368.00, *p* = 0.044 respectively) (Table 4). Conversely, salary and current smoking status significantly reduced the odds of low resilience amongst doctors (OR 0.13, 95%CI 0.02–0.64, *p* = 0.024 and OR 0.16, 95%CI 0.02–0.66, *p* = 0.027 respectively). In addition, for ambulance personnel and overall sample, only previous alcohol use significantly reduced the odds of low resilience (OR 0.44, 95%CI 0.20–0.94, *p* = 0.038 and OR 0.52, 95%CI 0.29–0.91, *p* = 0.024 respectively). It should also be noted that the results from the multivariable logistic analysis reported in Table 4 are consistent with the results from the bivariable analysis in Table 3.

**Table 3:** Comparison of CD-RISC-10 score across independent variables

		Doctors		Ambulance personnel		Overall		
**Variable**	**Group**	Mean*	P value*	Mean*	P value*	Mean*	P value*	
Gender	Female	**25.84**	**< 0.001** ^**a**^	28.29	0.595^a^	**27.16**	**0.035** ^**a**^	
	Male	**28.73**		27.79		**28.11**		
Age (*N* = 339)	20–29	26.53	0.337 ^b^	29.34	0.150 ^b^	27.51	0.309 ^b^	
	30–39	27.04		28.45		28.02		
	40–49	27.14		26.78		26.89		
	> 50	29.19		27.93		28.41		
Home language	English	27.22	0.748 ^b^	27.67	0.478 ^b^	27.41	0.152 ^b^	
	Afrikaans	27.50		28.47		28.24		
	IsiXhosa	25.90		27.54		27.10		
	Other	22.00		28.50		25.71		
Relationship Status	Married	27.80	0.143 ^b^	27.65	0.374 ^b^	27.71	0.743 ^b^	
	Never married	26.29		28.30		27.44		
	Divorced/Separated/Widowed	27.73		28.38		28.21		
Professional health qualification	Yes	27.06	N/A	27.92	0.775 ^a^	27.54	0.276 ^a^	
	No	N/A		28.48		28.48		
Job category	Operational services/EMS	N/A	0.159 ^b^	27.78	0.561 ^b^	**27.78**	**0.019** ^**b**^	
	Support staff/EMS	N/A		28.60		**28.60**		
	Junior doctors	26.40		N/A		**26.40**		
	Senior doctors	27.38		N/A		**27.38**		
Over-time work	Yes	26.98	0.257 ^a^	27.97	0.942 ^a^	27.49	0.186 ^a^	
	No	29.50		28.11		28.19		
Monthly Salary (ZAR)	R0 - R15 000	**N/A**	**0.020** ^**b**^	27.65	0.945 ^b^	27.65	0.054^b^	
	R15 001 - R30 000	**N/A**		28.22		28.22		
	R30 001- R50 000	**25.91**		28.73		26.63		
	> R50 001	**27.65**		N/A		27.65		
Smoking history	Never used	**26.65**	**0.012** ^**b**^	28.07	0.806 ^b^	27.39	0.079 ^b^	
	Previous smoker	**27.17**		27.17		27.17		
	Current smoker	**30.74**		28.16		28.58		
Alcohol history	Never used	26.67	0.618 ^b^	**27.11**	**0.002** ^**b**^	**26.96**	**0.020** ^**b**^	
	Previous alcohol user	26.59		**30.47**		**29.18**		
	Current drinker	27.30		**27.56**		**27.44**		
Illicit drug use	Never used	26.94	0.607 ^b^	28.02	0.431 ^b^	27.57	0.475 ^b^	
	Previous illicit drug user	28.00		28.34		28.25		
	Current illicit drug user	29.43		26.91		27.89		
Ever diagnosed with a mental health condition (*N* = 646)	Yes	**25.66**	**0.037** ^**a**^	**25.47**	**0.010** ^**a**^	**25.58**	**< 0.001** ^**a**^	
	No	**27.47**		**28.33**		**28.02**		
Currently on treatment for mental health condition	Yes	25.58	0.088 ^a^	**25.54**	**0.029** ^**a**^	**25.56**	**0.002** ^**a**^	
	No	27.37		**28.21**		**27.90**		
Feel need to smoke to manage WRS	Yes	28.44	0.194 ^a^	27.56	0.286 ^a^	27.83	0.765 ^a^	
	No	26.77		28.18		27.57		
Feel need to drink alcohol to manage WRS (*N* = 644)	Yes	25.36	**0.034** ^**a**^	**26.36**	**0.048** ^**a**^	**25.81**	**0.002** ^**a**^	
	No	27.45		**28.23**		**27.94**		
Feel need to use illicit drugs to manage WRS (*N* = 642)	Yes	26.00	0.488 ^a^	28.44	0.875 ^a^	27.34	0.570 ^a^	
	No	27.16		28.00		27.67		

* Statistically significant results are indicated in bold; ^a^ Mann–Whitney test; ^b^ Kruskal–Wallis test

EMS: Emergency medical services; N/A: not applicable; WRS: work-related stress; ZAR: South African Rand

**Table 4:** Multivariable logistic regression models for predictors of the CD-RISC-10 score

Predictors	OR (95%CI) ^*^			*P* value^*^		
	Doctors	Ambulance personnel	Overall	Doctors	Ambulance personnel	Overall
**Gender**						
Male	1.00	1.00	1.00			
Female	**1.94 (1.03–3.72)**	1.19 (0.70–2.03)	1.41 (0.95–2.08)	**0.043**	0.517	0.086
**Age**						
20–29	1.00	1.00	1.00			
30–39	0.97 (0.41–2.31)	1.19 (0.57–2.59)	1.03 (0.60–1.80)	0.946	0.653	0.913
40–49	0.94 (0.32–2.75)	1.68 (0.76–3.89)	1.27 (0.69–2.37)	0.914	0.210	0.446
> 50	0.69 (0.12–3.58)	1.08 (0.35–3.27)	0.86 (0.36–2.04)	0.659	0.897	0.739
**Home language**						
English	1.00	1.00	1.00			
Afrikaans	0.64 (0.30–1.31)	0.84 (0.48–1.46)	0.85 (0.56–1.29)	0.229	0.529	0.439
IsiXhosa	1.65 (0.67–4.13)	1.21 (0.61–2.37)	1.23 (0.74–2.04)	0.278	0.585	0.420
Other	0.93 (0.03–18.40)	2.21 (0.24–20.30)	1.33 (0.24–6.60)	0.957	0.452	0.729
**Job category**						
Operational services/ EMS		1.00	1.00			
Support staff/ EMS		0.71 (0.40–1.25)	0.68 (0.39–1.17)		0.244	0.167
Junior doctors	1.00		1.87 (0.64–5.91)			0.268
Senior doctors	**6.94 (1.22–60.50)**		4.92 (1.00-29.90)	**0.044**		0.061
**Years employed in current role**	1.02 (0.94–1.11)	1.04 (1.00-1.08)	1.03 (0.99–1.07)	0.668	0.080	0.118
**Overtime**						
No	1.00	1.00	1.00			
Yes	**13.88 (1.61–368.00)**	0.77 (0.46–1.30)	0.92 (0.57–1.49)	**0.044**	0.333	0.729
**Monthly Salary (ZAR)**						
R0 - R15,000		1.00	1.00			
R15 001-R30 000		0.77 (0.45–1.32)	0.87 (0.52–1.44)		0.348	0.582
R30 001-R50 000	1.00	0.55 (0.19–1.47)	0.65 (0.23–1.67)		0.251	0.391
> R50 001	**0.13 (0.02–0.64)**		0.18 (0.03–0.94)	**0.024**		0.052
**Smoking history**						
Never used	1.00	1.00	1.00			
Previous smoker	1.98 (0.70–5.61)	1.13 (0.48–2.58)	1.32 (0.70–2.45)	0.195	0.782	0.389
Current smoker	**0.16 (0.02–0.66)**	0.92 (0.50–1.67)	0.84 (0.51–1.38)	**0.027**	0.789	0.499
**Alcohol history**						
Never used	1.00	1.00	1.00			
Previous alcohol user	0.66 (0.25–1.69)	**0.44 (0.20–0.94)**	**0.52 (0.29–0.91)**	0.389	**0.038**	**0.024**
Current Drinker	0.51 (0.24–1.08)	1.36 (0.74–2.52)	0.91 (0.58–1.44)	0.080	0.322	0.678
**Illicit drug use**						
Never used	1.00	1.00	1.00			
Previous illicit drug user	0.67 (0.12–2.86)	0.63 (0.24–1.54)	0.68 (0.3–1.41)	0.607	0.336	0.313
Current illicit drug user	0.24 (0.01–1.98)	1.26 (0.28–5.05)	0.70 (0.20–2.09)	0.245	0.751	0.540
**Substance use to manage WRS**						
Feel need to drink alcohol to manage WRS	1.39 (0.66–2.94)	1.15 (0.52–2.45)	1.25 (0.75–2.08)	0.388	0.729	0.390
**Mental health**						
Ever diagnosed with a mental health condition	1.76 (0.61–5.24)	1.65 (0.68–3.95)	1.66 (0.87–3.15)	0.295	0.258	0.121
Currently on treatment for mental health condition	0.90 (0.27–2.9)	1.60 (0.56–4.48)	1.23 (0.59–2.55)	0.862	0.370	0.571

^*^Statistically significant results are indicated in bold

EMS: Emergency medical services; WRS: Work-related stress, ZAR/R: South African Rand

## Discussion

This study aimed to estimate the prevalence of resilience and determinants of psychological resilience among a group of healthcare workers in South Africa comprising doctors and ambulance personnel.

The study found the prevalence of psychological resilience among healthcare workers was relatively low, at 27.6 (± 6.6). The average score of the ambulance personnel (28.0 ± 6.9) was greater than that of the doctors (27.1 ± 6.0). Kang and colleagues reported an overall average score of 29.0 (± 6.8) for a group of ambulance personnel in China, which is higher than the overall average score obtained in this study [25]. A study comparing doctors and ambulance technicians in Spain, reported an overall average score of 30.6 (± 5.0), which was higher than that obtained in the present study [26]. A longitudinal study on healthcare workers in South Africa reported average scores of 26.7 (± 8.8) and 30 (± 6.7) for the two time points considered [3]. The average resilience score for the second time point of the longitudinal study was greater than that of the present study. Furthermore, two studies on Malaysian healthcare workers reported overall average scores of 28.6 (± 6.3) and 30.0 (± 6.3), respectively, both of which were higher than those in the present study [22, 27]. Zhou and colleagues, however, reported an overall average score of 23.2 (± 9.3) in their study of Chinese resident doctors, which is lower than that obtained in the present study [28]. This variability in the level of resilience observed may be due to differences in the study context (population sampled, time when the study was conducted), resources available in the healthcare system and differences in cultural values and norms, which may result in different coping styles among healthcare workers [5]. Overall, the results from this study were consistent with results from comparative studies on the resilience of healthcare workers when considering the standard deviations reported.

The study revealed a statistically significant association between psychological resilience and gender, with females having significantly lower resilience than males. These results are consistent with previous studies on psychological resilience showing that female gender is associated with lower resilience scores [12, 22, 29, 30]. This could be attributed to females assuming multiple roles at home and in the workplace, experiencing more emotional exhaustion and being more sensitive and susceptible to stress [12, 29]. The difference could also be due to social desirability bias, with males answering in a way that portrays an image of being able to manage pressure better [22].

We observed that doctors who were current smokers had greater average resilience scores than did those who were previous smokers and those who had never smoked before. These results contrast with the results of previous studies in which current smokers were found to have significantly lower psychological resilience [31]. It is probable that current smoking may be reflective of a coping mechanism and could mask low levels of resilience among current smokers. Substance use and medication use have been used as maladaptive coping mechanisms to address mental health issues and work-related stress [14, 32].

Similarly, in ambulance personnel and the overall sample, a significant relationship was found between psychological resilience and alcohol history, with previous alcohol users having reduced odds of low resilience. Guidelines for rehabilitation programs (alcohol and smoking) consider improving resilience to be necessary for preventing substance use onset, abuse problems and relapse [31, 33, 34]. In addition, Yamashita and colleagues reported that a lower relapse risk was associated with greater resilience [35]. It is also probable that previous alcohol use may be reflective of a coping mechanism and could mask low levels of resilience among previous alcohol users.

This study found no significant associations between psychological resilience and other sociodemographic or lifestyle factors, such as age, home language and relationship status. This is consistent with the results of previous research on resilience [18, 36, 37].

Years in the current role and professional qualifications were not found to be significant predictors of the CD-RISC-10 score in the present study. Wang and colleagues argued that senior healthcare workers have better experience and professional skills to address complex situations that arise in the workplace [21]. Previous researchers have reported that years in practice was positively associated with psychological resilience [20, 23]. Afshari and colleagues noted that an increase in healthcare workers’ education and work experience may be linked to the progression of skills, which results in the development of positive coping strategies, leading to greater resilience [38]. Herman and colleagues noted that these inconsistencies observed between psychological resilience and predictive factors may be due to differences in study methodologies and the definition of resilience used by the investigators [9].

Notably, the average resilience of ambulance personnel was significantly greater than that of doctors in this study, similar to the findings of Mantas-Jiménez and colleagues, who compared doctors and ambulance technicians in Spain [26]. This could be attributable to the social demographic and work-related characteristics of ambulance personnel compared to doctors in the study. Ambulance personnel were older and mostly male, had longer years of service and worked less overtime compared to the doctors. Organisational factors such as the culture within the ambulance service could be different to the medical hospital-based environment. These factors have all been reported previously as factors associated with higher resilience for healthcare workers [11].

Overtime work was found to be significant negatively associated with resilience among doctors in the present study. These results are in line with the interventions recommended by the healthcare workers in the present study to reduce WRS, with most of the participants indicating that addressing staff shortages was important for reducing WRS. A study on nurses in China, also found that working longer hours a day resulted in significantly lower psychological resilience [39]. However, Rossouw and colleagues did not find any significant relationship between resilience and overtime hours in their study of healthcare workers in South Africa [18]. High workload and occupational stressors were likely to lead to low job satisfaction, poor work performance and high job turnover for healthcare workers, resulting in a vicious cycle and ultimately leading to burnout and low resilience [30].

The present study revealed a significantly negative association between psychological resilience and self-reported mental health conditions and treatment for mental health conditions for the overall sample. Past research on resilience has found that psychological resilience has been identified to have a protective role against mental health issues [40, 41]. A study on Indonesian medical students, reported that higher resilience was moderately correlated with lower scores for depressive and anxious symptoms [42]. In addition, Keragholi and colleagues, in their study of Iranian ambulance personnel, also reported that mental health status was negatively associated with resilience [40]. A study on South African healthcare workers reported that healthcare workers using medication or other forms of treatment for their anxiety or depression symptoms had significantly lower resilience than did those not using medication [18]. Furthermore, stigma and denial related to mental health might impact the ability of healthcare workers to seek help, which could also lead to underreporting in research studies [18].

The resilience score of participants who reported needing to use alcohol to manage WRS was significantly lower than that of participants who reported not needing to use alcohol. In addition, the preference of most participants (76.7%) was for the provision of psychological counselling as an intervention that could be provided by institutions to assist with reducing WRS. This is a positive coping strategy compared to substance use, which is recognised as a maladaptive coping mechanism used by those with mental health issues or WRS [32]. In addition, resilience interacts with stress to impact on the development of addiction and relapse [33]. Other studies have also identified the protective role of psychological resilience on WRS [43].

### Strengths and limitations

The primary strength of this study was that it included a large population of healthcare workers in South Africa. In addition, both previous surveys used to collect data for this study had good response rates. The study also used a validated and standardised questionnaire to measure the outcome variable, which provides an opportunity to compare the results of this study with those of previous studies.

This study had several limitations. First, as a secondary data analysis was undertaken, the information available was limited to what had been provided and collected from the previous two studies. Second, causation cannot be inferred via a cross-sectional study design, and the risk factors identified need to be interpreted accordingly. Third, as self-reported data were used, the risk of social desirability bias was high, as respondents may have been influenced by stigma associated with substance use and mental health. In addition, recall bias may have occurred during the initial data collection phase where the participants’ memory was relied upon. Most questions used in this study, however, did not require recall over many months. Fourth, selection bias was largely unavoidable, as participation in the surveys was voluntary, and those who had been experiencing problems such as PTSD or burnout may have been more likely to complete the survey, as PTSD and burnout were the focus of the primary studies. In addition, confidentiality concerns may also affect participation and contribute to bias. The initial investigators had put in place measures to mitigate this bias, including introductory letters to explain the data handling procedure and the preservation of confidentiality. Last, the healthy worker effect may result in the overestimation of healthcare workers’ resilience status since those with low levels of resilience may have already left active work.

## Conclusion and recommendations

Resilience was relatively low in this group of South African healthcare workers compared to similar studies globally, highlighting the need to build resilience among healthcare workers in South Africa. This study demonstrated that resources need to be directed towards building resilience among female healthcare workers, those working long hours and earning lower income. In addition, support such as psychological counselling should be offered to healthcare workers who have been diagnosed with mental health conditions. Further research is needed to better characterise the sociodemographic and work-related factors impacting the psychological resilience of healthcare workers in South Africa. Additional research could focus on resilience specifically, consider a larger and more representative sample and include qualitative research methods. This will assist in understanding determinants of psychological resilience and may inform intervention strategies that would build psychological resilience in the healthcare workforce in South Africa.

### Electronic supplementary material

Below is the link to the electronic supplementary material.

Supplementary Material 1

## Data Availability

The data are available upon reasonable request from the corresponding author.

## Abbreviations

CART: Classification and regression tree
CD-RISC: Connor-Davidson Resilience Scale
CD-RISC-10: Connor-Davidson Resilience Scale 10
CD-RISC-25: Connor-Davidson Resilience Scale 25
95%CI: 95% Confidence Interval
COVID-19: Coronavirus disease
EMS: Emergency medical services
HCWs: Healthcare Workers
HICs: High-income countries
HIV/AIDS: Human Immunodeficiency Virus/Acquired Immune Deficiency Syndrome
IQR: Interquartile Range
LMICs: Low-and middle-income countries
m: Mean
MICE: Multivariate Imputation by Chained Equation
N: Number
N/A: Not applicable
NHI: National Health Insurance
OR: Odds ratio
p/ p value: Probability Value
PTSD: Posttraumatic stress disorder
SD: Standard deviation
WRS: Work-Related Stress
ZAR/R: South African Rand
