# Supplementary material for: Sociodemographic and work-related factors associated with psychological resilience in South African healthcare workers: a cross-sectional study
Source: BMC Health Serv Res. 2024 Aug 24;24:979. doi: 10.1186/s12913-024-11430-0 (PMC11344366; doi:10.1186/s12913-024-11430-0)
Supplement: Supplementary file 1 — Supplementary Material 1 [file 12913_2024_11430_MOESM1_ESM.pdf]

## SUPPLEMENTARY INFORMATION

**Supplementary Table S1: Summary of missing data in the original dataset**

| **Missing data:** | **N (%)** |
|---|---|
| Age | 49 (7.6%) |
| Years employed in current role | 5 (0.8%) |
| Current smoker | 2 (0.3%) |
| Current drinker | 1 (0.2%) |
| Current illicit drug user | 1 (0.2%) |
| Ever diagnosed with a mental health condition | 1 (0.2%) |
| Feel need to use drink to manage WRS | 3 (0.5%) |
| Feel need to use illicit drugs to manage WRS | 5 (0.8%) |

The range of missingness in the dataset is between 0.2% and 7.6%. Age was the only factor with greater than 1% of the data missing; as such, it was the only factor for which imputation was performed.

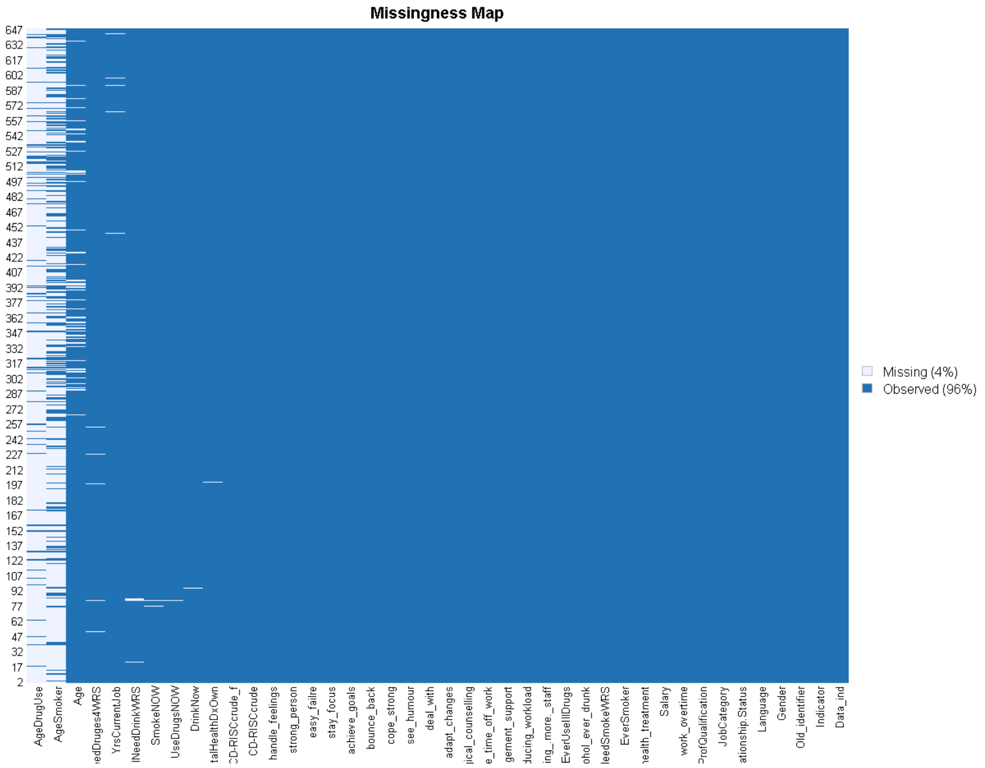


**Supplementary Fig. S1: Missingness map of the dataset**

**Supplementary Table S2: Summary statistics of Age covariable following imputation**

| | Missing values | Minimum | First quartile | Median | Mean | Third quartile | Maximum |
|---|---|---|---|---|---|---|---|
| Original | 49 | 1 | 1 | 2 | 2.216 | 3 | 5 |
| Predictive mean matching | 0 | 1 | 1 | 2 | 2.234 | 3 | 5 |
| Classification and regression tree | 0 | 1 | 1 | 2 | 2.232 | 3 | 5 |
| Lasso linear regression | 0 | 0.574 | 1 | 2 | 2.401 | 3 | 5 |

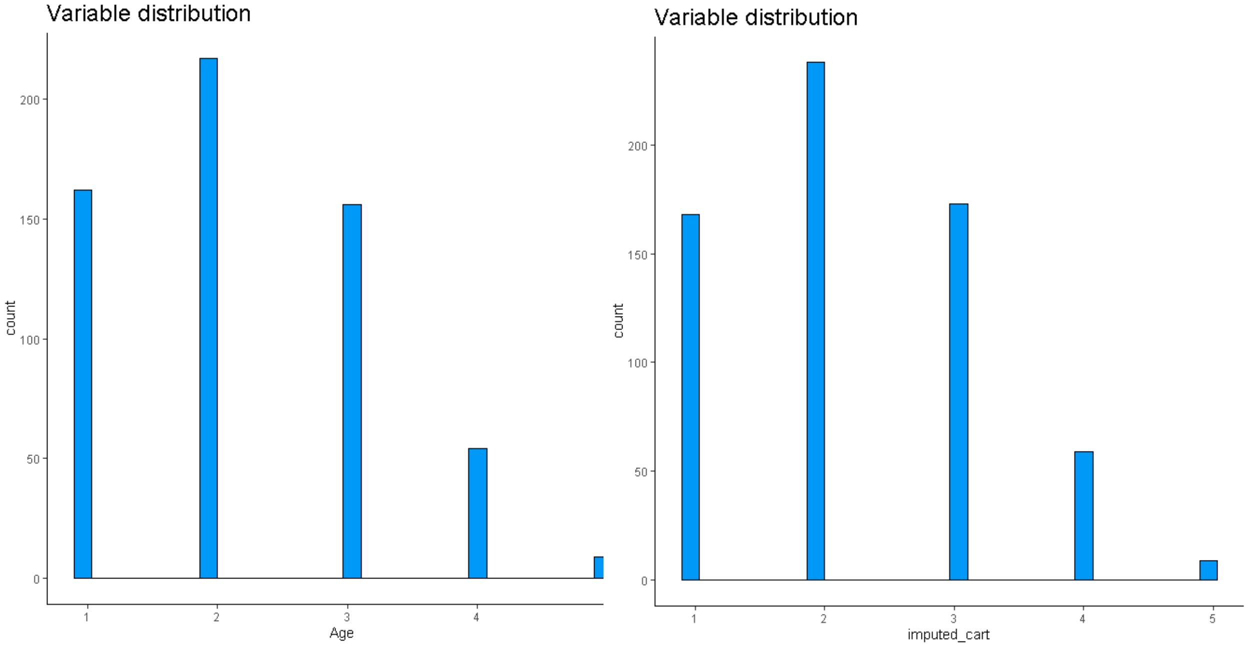


**Supplementary Fig. S2: Distribution of Age factor before and after CART imputation**

Supplementary Table S3: Unadjusted logistic regression analysis of the predictors of CD-RISC-10 score (all variables)

| | OR (95% CI) * | | | P value * | | |
|---|---|---|---|---|---|---|
| **Variables** | **Doctors** | **Ambulance personnel** | **Overall** | **Doctors** | **Ambulance personnel** | **Overall** |
| **Gender** | | | | | | |
| Male | 1.00 | 1.00 | 1.00 | | | |
| Female | **2.45 (1.44-4.26)** | 0.92 (0.60-1.42) | **1.40 (1.01- 1.95)** | **0.001** | 0.711 | **0.045** |
| **Age** | | | | | | |
| 20-29 | 1.00 | 1.00 | 1.00 | | | |
| 30-39 | 0.99 (0.53-1.82) | 1.30 (0.66-2.65) | 0.94 (0.62- 1.44) | 0.969 | 0.459 | 0.782 |
| 40-49 | 0.95 (0.47-1.89) | 1.89 (0.95-3.92) | 1.20 (0.77- 1.88) | 0.877 | 0.078 | 0.424 |
| >50 | 0.75 (0.29-1.83) | 1.39 (0.57-3.39) | 0.91 (0.49- 1.65) | 0.535 | 0.463 | 0.751 |
| **Home language** | | | | | | |
| English | 1.00 | 1.00 | 1.00 | | | |
| Afrikaans | 0.81 (0.41-1.53) | 0.84 (0.51-1.4) | 0.77 (0.53- 1.11) | 0.522 | 0.498 | 0.165 |
| IsiXhosa | 1.45 (0.66-3.14) | 1.23 (0.68-2.21) | 1.20 (0.76- 1.87) | 0.348 | 0.494 | 0.432 |
| Other | 0.88 (0.04-9.36) | 2.21 (0.26-19.00) | 1.45 (0.28- 6.69) | 0.917 | 0.436 | 0.633 |
| **Relationship Status** | | | | | | |
| Married | 1.00 | 1.00 | 1.00 | | | |
| Never married | 1.56 (0.92-2.66) | 0.82 (0.52-1.29) | 1.09 (0.77- 1.53) | 0.100 | 0.389 | 0.638 |
| Divorced/ Separated/ Widowed | 1.50 (0.47-4.48) | 0.92 (0.43-1.87) | 1.06 (0.57- 1.93) | 0.472 | 0.822 | 0.839 |
| **Professional health qualification** | | | | | | |
| No | 1.00 | 1.00 | 1.00 | | | |

| | | | | | | |
|---|---|---|---|---|---|---|
| Yes | N/A | 1.22 (0.69-2.25) | 1.35 (0.78- 2.43) | N/A | 0.512 | 0.304 |
| | | | | | | |
| **Job category** | | | | | | |
| Operational services/ EMS | | 1.00 | 1.00 | | | |
| Support staff/ EMS | | 0.69 (0.42-1.12) | 0.69 (0.42- 1.12) | | 0.142 | 0.142 |
| Junior doctors | 1.00 | | 1.23 (0.74- 2.04) | | | 0.415 |
| Senior doctors | 0.92 (0.54-1.58) | | 1.13 (0.76- 1.68) | 0.752 | | 0.544 |
| | | | | | | |
| **Years employed in current role** | 1.00 (0.95-1.04) | 1.03 (1.00-1.07) | 1.01 (0.99- 1.04) | 0.832 | 0.056 | 0.379 |
| | | | | | | |
| **Over-time work** | | | | | | |
| No | 1.00 | 1.00 | 1.00 | | | |
| Yes | 4.12 (0.72-77.60) | 0.92 (0.58-1.46) | 1.13 (0.75- 1.73) | 0.189 | 0.708 | 0.559 |
| | | | | | | |
| **Monthly Salary (ZAR)** | | | | | | |
| R0-R15 000 | | 1.00 | 1.00 | | | |
| R15 001-R30 000 | | 0.84 (0.54-1.31) | 0.84 (0.54- 1.31) | | 0.44 | 0.44 |
| R30 001-R50 000 | 1.00 | 0.61 (0.23-1.44) | 1.15 (0.70- 1.88) | | 0.283 | 0.588 |
| > R50 001 | 0.74 (0.44-1.26) | | 1.03 (0.65- 1.62) | 0.268 | | 0.910 |
| | | | | | | |
| **Age started smoking** | 1.01 (0.93-1.09) | 1.01 (0.93-1.09) | 1.01 (0.94- 1.08) | 0.812 | 0.812 | 0.729 |
| **Age started illicit drugs** | 1.24 (0.91-1.81) | 1.05 (0.95-1.16) | 1.07 (0.97- 1.17) | 0.193 | 0.315 | 0.171 |
| | | | | | | |
| **Smoking history** | | | | | | |
| Never used | 1.00 | 1.00 | 1.00 | | | |
| Previous smoker | 1.23 (0.50-2.92) | 1.16 (0.53-2.41) | 1.16 (0.65- 2.02) | 0.642 | 0.702 | 0.617 |
| Current smoker | **0.15 (0.02-0.54)** | 0.90 (0.55-1.45) | **0.65 (0.42- 0.98)** | **0.012** | 0.664 | **0.046** |

| | | | | | | |
|---|---|---|---|---|---|---|
| **Alcohol history** | | | | | | |
| Never used | 1.00 | 1.00 | 1.00 | | | |
| Previous alcohol user | 0.73 (0.31-1.67) | **0.40 (0.19-0.79)** | **0.52 (0.30- 0.87)** | 0.452 | **0.011** | **0.015** |
| Current drinker | 0.56 (0.30-1.05) | 1.04 (0.64-1.71) | 0.83 (0.57- 1.22) | 0.069 | 0.878 | 0.349 |
| **Illicit drug use** | | | | | | |
| Never used | 1.00 | 1.00 | 1.00 | | | |
| Previous illicit drug user | 0.50 (0.11-1.67) | 0.65 (0.27-1.42) | 0.58 (0.28- 1.12) | 0.297 | 0.305 | 0.124 |
| Current Drug user | 0.28 (0.01-1.65) | 1.26 (0.32-4.25) | 0.75 (0.24- 2.02) | 0.237 | 0.722 | 0.589 |
| **Substance use to manage WRS** | | | | | | |
| Feel need to smoke to manage WRS | 0.67 (0.32-1.32) | 1.16 (0.71-1.87) | 0.93 (0.63- 1.38) | 0.258 | 0.548 | 0.731 |
| Feel need to drink alcohol to manage WRS | 1.32 (0.71-2.44) | 1.67 (0.86-3.16) | 1.53 (0.98- 2.38) | 0.379 | 0.121 | 0.060 |
| Feel need to use illicit drugs to manage WRS | 1.11 (0.33-3.42) | 1.03 (0.32-2.9) | 1.08 (0.47- 2.32) | 0.863 | 0.959 | 0.850 |
| **Mental health** | | | | | | |
| Ever diagnosed with a mental health condition | 1.73 (0.97-3.06) | 1.74 (0.90-3.31) | **1.81 (1.18- 2.75)** | 0.061 | 0.094 | **0.006** |
| Currently on treatment for mental health condition | 1.51 (0.78-2.90) | 1.77 (0.80-3.86) | **1.69 (1.03- 2.77)** | 0.213 | 0.151 | **0.037** |

* Statistically significant results are indicated in bold

N/A: not applicable; ZAR/R: South African Rand

**Supplementary Table S4: Adjusted logistic regression analysis of the predictors of CD-RISC-10 score (all variables)**

| Variables | OR (95% CI) †* | | | P value †* | | |
|---|---|---|---|---|---|---|
| | Doctors | Ambulance personnel | Overall | Doctors | Ambulance personnel | Overall |
| **Home language** | | | | | | |
| English | 1.00 | 1.00 | 1.00 | | | |
| Afrikaans | 0.78 (0.39-1.51) | 0.83 (0.50-1.38) | 0.76 (0.52- 1.11) | 0.477 | 0.467 | 0.153 |
| IsiXhosa | 1.51 (0.67-3.36) | 1.31 (0.72-2.38) | 1.18 (0.75- 1.85) | 0.310 | 0.384 | 0.475 |
| Other | 0.80 (0.04-9.04) | 2.21 (0.25-19.40) | 1.58 (0.30- 7.40) | 0.858 | 0.442 | 0.561 |
| **Relationship Status** | | | | | | |
| Married | 1.00 | 1.00 | 1.00 | | | |
| Never married | 1.39 (0.73-2.69) | 0.98 (0.59-1.65) | 1.10 (0.74- 1.63) | 0.318 | 0.948 | 0.653 |
| Divorced/ Separated/ Widowed | 1.19 (0.36-3.72) | 0.99 (0.46-2.07) | 1.00 (0.54- 1.83) | 0.773 | 0.980 | 0.991 |
| **Professional health qualification** | | | | | | |
| No | 1.00 | 1.00 | 1.00 | | | |
| Yes | N/A | 1.09 (0.60-2.06) | 1.40 (0.80- 2.56) | N/A | 0.784 | 0.254 |
| **Job category** | | | | | | |
| Operational services/ EMS | | 1.00 | 1.00 | | | |
| Support staff/ EMS | | 0.73 (0.43-1.22) | 0.65 (0.39- 1.07) | | 0.237 | 0.099 |
| Junior doctors | 1.00 | | 1.32 (0.69- 2.53) | | | 0.394 |
| Senior doctors | 0.99 (0.45-2.17) | | 1.09 (0.72- 1.64) | 0.982 | | 0.679 |
| **Years employed in current role** | 1.00 (0.93-1.08) | 1.03 (0.99-1.07) | 1.02 (0.99- 1.05) | 0.930 | 0.184 | 0.284 |

| | | | | | | |
|---|---|---|---|---|---|---|
| **Over-time work** | | | | | | |
| No | 1.00 | 1.00 | 1.00 | | | |
| Yes | 0.21 (0.01-1.27) | 0.96 (0.60-1.56) | 1.13 (0.74- 1.74) | 0.155 | 0.879 | 0.582 |
| **Monthly Salary (ZAR)** | | | | | | |
| R0-R15 000 | | 1.00 | 1.00 | | | |
| R15 001-R30 000 | | 0.77 (0.48-1.22) | 0.83 (0.52- 1.30) | | 0.266 | 0.409 |
| R30 001-R50 000 | 1.00 | 0.54 (0.19-1.35) | 1.22 (0.71- 2.09) | | 0.206 | 0.475 |
| > R50 001 | 0.72 (0.36-1.44) | | 1.01 (0.64- 1.61) | 0.358 | | 0.950 |
| **Age started smoking** | 1.11 (0.90-1.39) | 1.01 (0.93-1.08) | 1.01 (0.94- 1.08) | 0.315 | 0.897 | 0.782 |
| **Age started illicit drugs** | 1.36 (0.90-2.80) | 1.05 (0.94-1.18) | 1.07 (0.97- 1.20) | 0.231 | 0.403 | 0.195 |
| **Smoking history** | | | | | | |
| Never used | 1.00 | 1.00 | 1.00 | | | |
| Previous smoker | 1.68 (0.66-4.25) | 1.06 (0.48-2.27) | 1.26 (0.69- 2.24) | 0.273 | 0.873 | 0.442 |
| Current smoker | **0.21 (0.03-0.77)** | 0.91 (0.55-1.49) | 0.77 (0.45- 1.09) | **0.042** | 0.714 | 0.127 |
| **Alcohol history** | | | | | | |
| Never used | 1.00 | 1.00 | 1.00 | | | |
| Previous alcohol user | 0.78 (0.32-1.84) | **0.39 (0.18-0.78)** | **0.52 (0.3- 0.88)** | 0.568 | **0.010** | 0.015 |
| Current drinker | 0.64 (0.33-1.23) | 1.12 (0.68-1.88) | 0.91 (0.62- 1.36) | 0.18 | 0.657 | 0.654 |
| **Illicit drug use** | | | | | | |
| Never used | 1.00 | 1.00 | 1.00 | | | |
| Previous illicit drug user | 0.73 (0.16-2.61) | 0.64 (0.26-1.42) | 0.63 (0.30- 1.25) | 0.651 | 0.296 | 0.206 |
| Current Drug user | 0.31 (0.02-1.91) | 1.24 (0.32-4.22) | 0.76 (0.24- 2.07) | 0.283 | 0.736 | 0.611 |

| | | | | | | |
|---|---|---|---|---|---|---|
| **Substance use to manage WRS** | | | | | | |
| Feel need to smoke to manage WRS | 0.76 (0.36-1.54) | 1.21 (0.73-1.97) | 1.00 (0.66- 1.48) | 0.449 | 0.456 | 0.98 |
| Feel need to drink alcohol to manage WRS | 1.17 (0.61-2.20) | 1.73 (0.89-3.33) | 1.56 (0.99- 2.43) | 0.628 | 0.100 | 0.053 |
| Feel need to use illicit drugs to manage WRS | 1.07 (0.31-3.41) | 1.06 (0.33-3.00) | 1.07 (0.47- 2.30) | 0.909 | 0.921 | 0.872 |
| | | | | | | |
| **Mental health** | | | | | | |
| Ever diagnosed with a mental health condition | 1.56 (0.87-2.81) | 1.75 (0.90-3.35) | **1.77 (1.15- 2.70)** | 0.136 | 0.095 | **0.009** |
| Currently on treatment for mental health condition | 1.38 (0.70-2.69) | 1.80 (0.80-3.95) | **1.70 (1.03- 2.80)** | 0.348 | 0.145 | **0.037** |

* Statistically significant results are indicated in bold; † Data adjusted for age and gender

N/A: not applicable; ZAR/R: South African Rand
